# Adhesion and Proliferation of Human Periodontal Ligament Cells on Poly(2-methoxyethyl acrylate)

**DOI:** 10.1155/2014/102648

**Published:** 2014-08-06

**Authors:** Erika Kitakami, Makiko Aoki, Chikako Sato, Hiroshi Ishihata, Masaru Tanaka

**Affiliations:** ^1^Graduate School of Science and Engineering, Yamagata University, Jonan 4-3-16, Yonezawa, Yamagata 992-8510, Japan; ^2^Graduate School of Dentistry, Division of Periodontology and Endodontology, Tohoku University, Seiryo-machi 4-1, Sendai, Miyagi 980-8575, Japan

## Abstract

Human periodontal ligament (PDL) cells obtained from extracted teeth are a potential cell source for tissue engineering. We previously reported that poly(2-methoxyethyl acrylate) (PMEA) is highly biocompatible with human blood cells. In this study, we investigated the adhesion, morphology, and proliferation of PDL cells on PMEA and other types of polymers to design an appropriate scaffold for tissue engineering. PDL cells adhered and proliferated on all investigated polymer surfaces except for poly(2-hydroxyethyl methacrylate) and poly[(2-methacryloyloxyethyl phosphorylcholine)*-co-*(*n*-butyl methacrylate)]. The initial adhesion of the PDL cells on PMEA was comparable with that on polyethylene terephthalate (PET). In addition, the PDL cells on PMEA spread well and exhibited proliferation behavior similar to that observed on PET. In contrast, platelets hardly adhered to PMEA. PMEA is therefore expected to be an excellent scaffold for tissue engineering and for culturing tissue-derived cells in a blood-rich environment.

## 1. Introduction

Periodontal diseases, caused by the bacterial biofilm, can affect up to 90% of adults worldwide [1]. Severe periodontitis leads to losing connective tissue and bone support and finally losing teeth [1]. Therefore, much research is being conducted on periodontal tissue regeneration for restoring the alveolar support of the teeth. The periodontal ligament (PDL) is an important structure that is composed of periodontal tissue, in which PDL cells generate connective tissue fibers that span the gap between the cementum and the alveolar bone [2] to suspend the tooth. This complex structure of PDL tissue comprises several different cell populations [3], including PDL cells, which are predominantly fibroblasts and play crucial roles in maintaining and regenerating periodontal tissue [2, 3].

Human PDL cells obtained from extracted teeth are a promising cell source for periodontal tissue regeneration, regenerative medicine, and tissue engineering [3–6], because they include stem cells that have a high capacity for proliferation, self-renewal, and multilineage differentiation and also have the ability to form cementum/periodontal ligament-like tissue* in vivo *[3, 4]. Human PDL stem cells, similar to bone marrow mesenchymal stem cells, are able to suppress immune responses and inflammatory reactions, which suggests that PDL stem cells may be used in allogeneic stem cell-based therapies [5]. In the first clinical application of human autologous PDL-derived cells, including PDL stem cells, the transplanted cells were used to reconstruct periodontal intrabony defects in 3 patients, and a significant improvement of periodontal disease was achieved, which suggests that PDL stem cell transplantation may be an efficacious and safe alternative for the treatment of human periodontitis [6].

Furthermore, human induced pluripotent stem cells (iPS cells) have been generated from adult human periodontal ligament fibroblasts [7]. Nomura et al. reported that the reprogramming efficiency of human PDL fibroblasts was 0.025%, which is not lower than the reprogramming efficiency of dental stem cells, even though the stem cells already express a number of ES cell-associated genes; therefore, human PDL fibroblasts may be an optimal cell source for generating iPS cells [7]. Additionally, the use of PDL cells is advantageous because it efficiently utilizes extracted teeth. However, for periodontal tissue regeneration, it is necessary to culture PDL cells on a biocompatible scaffold to deliver the cells to the wound site and also to provide space for the formation of the new periodontal tissue. Biomaterial scaffolds designed for tissue-engineered constructs must accommodate cell viability, growth, and function [8]. There is increasing interest in designing new biomaterials for scaffolds with minimal or no immune response that encourage stable implant/tissue interaction [9]. In addition, the surface characterization of biomaterials is important to design new implantable materials [10–14].

We previously reported that poly(2-methoxyethyl acrylate) (PMEA) shows excellent biocompatibility with human blood coagulation and complement systems and does not activate leukocytes, erythrocytes, and platelets* in vitro* and* ex vivo* relative to other polymer surfaces during the early stages of immune reactions [15–19]. On the basis of our results, superior biocompatible catheters and oxygenators coated with PMEA were approved by the Food and Drug Administration (FDA) and made available to the global market [16–19]. We further determined why PMEA has excellent biocompatibility [15, 20–22]. In particular, the low extent of platelet adhesion and spreading observed was closely related to a low degree of denaturation and high dissociation rate for proteins adsorbed onto PMEA [15].

The objective of this study was to examine the hypothesis that PMEA is a biocompatible polymer for tissue engineering that can facilitate adhesion and proliferation of PDL cells with low platelet adhesion. Cell-material interactions determine many cellular processes such as adhesion, spreading, and proliferation and are thus essential for tissue engineering [23–27]. However, the influence of the chemical components of synthesized polymers on the biology of PDL cells remains unclear. To investigate PDL cell-material interactions, we characterized the localization of focal adhesions, which are multifunctional organelles that mediate cell-material adhesion, force transmission, and cytoskeletal regulation and signaling [28]. To analyze the formation of focal adhesions, we evaluated the localization of vinculin, which is a membrane-cytoskeletal protein present in focal adhesions that is involved in the linkage of integrin adhesion molecules to the actin cytoskeleton [29].

## 2. Material and Methods

### 2.1. Preparation of Polymer Surfaces

PMEA was prepared by free-radical polymerization using 2,2′-azobisisobutyronitrile (Kanto Chemical Co., Inc., Japan) as the initiator and 2-methoxyethyl acrylate (MEA) as the monomer. The MEA was obtained from Wako Pure Chemical Industries, Ltd. (Osaka, Japan), poly(2-hydroxyethyl methacrylate) (PHEMA) was obtained from Scientific Polymer Products, Inc. (Ontario, NY), and poly[(2-methacryloyloxyethyl phosphorylcholine)-*co*-(*n*-butyl methacrylate)] (PMPC) was obtained from NOF corporation (Tokyo, Japan). The molecular weight of each polymer was estimated by gel permeation chromatography using polystyrene standards. The molecular weight (Mw) of PMEA, PHEMA, and PMPC was 85,000, 300,000, and 600,000, respectively. The chemical structure of each polymer PMEA, PHEMA, and PMPC is shown in Figure 1 in Supplementary Material available online at http://dx.doi.org/10.1155/2014/102648, and the polymers were prepared on polyethylene terephthalate (PET film: T100E125; Mitsubishi Plastics, Tokyo, Japan; 14 mm diameter and 125 *μ*m thickness) using a spin coater (MS-A100; Mikasa, Tokyo, Japan). Exactly 40 *μ*L of a 0.2 wt% solution of each polymer was cast twice onto the PET films. Analytical grade methanol (Kanto Chemical Co., Inc) was used as the solvent for each solution. The surfaces of the polymer films were analyzed by X-ray photoelectron spectroscopy (XPS) (ESCA-1000; Shimadzu, Kyoto, Japan) to confirm the coverage of the coated polymer. The take-off angle was 45°.

For cell culture, the films were sterilized by UV exposure for 2 h. Subsequently, the films were soaked in medium composed of Minimum Essential Medium (MEM-Alpha; Life Technologies) containing 10% heat-inactivated fetal bovine serum (Equitech-Bio, Inc., Kerrville, TX) and antibiotic solution (100 U/mL penicillin G sodium, 100 *μ*g/mL streptomycin sulfate, and 0.25 *μ*g/mL amphotericin B; Life Technologies) (culture medium) for one hour (preconditioning).

### 2.2. Characterization of Polymer Films

We analyzed each polymer film by atomic force microcopy (AFM; Agilent Technologies 5550 Scanning Probe Microscope, Agilent Technologies, Inc., Santa Clara, CA). The maximum scan range was approximately 10 *μ*m × 10 *μ*m using a cantilever with a force constant of 21–78 N/m, resonance frequency of 250–390 kHz, and tip height of 10–15 *μ*m (NCH-10, Nano World, Zurich, Switzerland). AFM was performed in air acoustic AC mode. AFM image analysis was performed using Pico Image Software (Agilent Technologies).

The wettability of the polymer surfaces was characterized by contact angle measurement [15]. The static contact angle on each polymer surface was measured using the sessile drop method at room temperature. For the sessile drop method, 2 *μ*L of deionized water was dropped on a dried polymer film using a microsyringe. The static contact angle was observed 30 s later under a microscope (G-1-1000; ERMA Inc., Tokyo, Japan). After at least five readings which were obtained for different areas of the polymer, the measurements were averaged to arrive at a final contact angle (*n* = 6).

### 2.3. Cell Preparation and Culture

Primary PDL cells were obtained as previously reported [30]. Fibroblast-like PDL cells were derived from the periodontal ligament of human third molars extracted from healthy individuals aged 17–21 years who had no clinical signs of chronic periodontal disease. Informed consent was obtained prior to each extraction. The cells were obtained from the Dental Faculty of Tohoku University. Periodontal ligament tissues were dissected into small pieces from the midportion of the root with a sharp blade. The pieces were then cultivated in tissue culture dishes (Asahi Glass Co., LTD, Tokyo, Japan) until the formation of a confluent cell monolayer using culture medium. After confluence was achieved, the cells were washed with phosphate-buffered saline (PBS; Takara Bio Inc., Shiga, Japan) and resuspended with 0.075 g/L protease and 0.1 g/L EDTA to enable passage. These experiments were approved by the Ethics Committee of the Dental Faculty of Tohoku University and the Graduate School of Science and Engineering of Yamagata University, Japan.

We used PDL cells at passages six and eight for adhesion and proliferation assays. PET, PMEA, PHEMA and PMPC films were put in 24-well polystyrene plates (Asahi Glass Co., LTD). After preconditioning of these films, PDL cells were seeded at 1 x 10^4^ cells/cm^2^ onto the tested films, and grown for up to 1 hour (1 h), 1 day, 3 days, and 7 days using culture medium. During culturing, the cells were maintained at 37°C in 5% CO_2_ and 95% air, and the medium was changed every three days. The progression of the cultures was examined by using phase contrast microscopy (CKX41; Olympus, Tokyo, Japan).

### 2.4. Immunofluorescence Staining

The adhesion, proliferation, and focal adhesion formation of the cultured PDL cells were observed by confocal laser scanning microscopy (CLSM; FV-1000; Olympus). To visualize cell adhesion, spreading, proliferation, and focal adhesion formation on the polymers, staining of vinculin, actin fibers, and cell nuclei was performed. After culture for the indicated period, the cells were washed with PBS twice. After washing, the cells were fixed with PBS containing 4% paraformaldehyde obtained from Wako Pure Chemical Industries, Ltd. (Osaka, Japan) for 10 min at 37°C and washed again three times with PBS. Subsequently, the cells were permeated three times with 1% Triton-X-100 (MP Biomedicals, LLC, Solon, OH) in PBS for 10 min at room temperature and then immersed in 0.02% Tween-PBS (MP Biomedicals, LLC) three times for 10 min each. To assess PDL cell-material interactions, PDL cells on polymers were stained for vinculin, which is localized at focal adhesions, using a mouse antivinculin monoclonal antibody (Millipore, Temecula, CA) as a primary antibody for 1 h, followed by treatment with Alexa Fluor 546 goat anti-mouse IgG (Life Technologies as a secondary antibody for 1 h. For actin staining, the samples were incubated with Alexa Fluor 488 phalloidin (Life Technologies) for 1 h. For detection of Ki-67 antigen as a cell proliferation marker, the samples were treated with rabbit anti-Ki-67 antigen monoclonal antibody (Life Technologies) for 2 h. The samples were then incubated with Alexa Fluor 488 goat anti-rabbit antibody (Life Technologies) for 1 h (*n* = 9). The stained cells were rinsed three times with PBS and subsequently immersed in PBS for 10 min. All specimens were placed on glass slides, mounted by using ProLong Gold antifade regent with DAPI (Life Technologies), and covered with glass cover slips.

The specimens were imaged by CLSM, and cell morphology parameters were quantified by Olympus Fluoview software. The total number of adherent cells on polymer films was counted in five randomly selected CLSM images (*n* = 3).

### 2.5. Scanning Electron Microscopy

To assess the morphology of adherent PDL cells cultured on each polymer for 1 h, the cells were observed by field emission scanning electron microscopy (SEM; SU-8000, Hitachi, Ltd., Tokyo, Japan). Cultured cells were fixed with 2.5% glutaraldehyde (Polysciences, Inc., Warrington, PA) in PBS and incubated overnight at 4°C. They were next washed three times with PBS and then with pure water and subsequently air-dried. The dried samples were coated with carbon using an ion sputter coater (HPC-1SW; Vacuum Device Inc., Ibaraki, Japan).

### 2.6. Platelet Adhesion Test

To investigate the number of platelets adhering to the polymers, blood was drawn from 3 healthy volunteers (nonsmokers; age 22 male, age 33 female, and age 42 male) and mixed with a 1/9 volume of 3.2% sodium citrate. Platelet-rich plasma (PRP) and platelet-poor plasma (PPP) were obtained by centrifugation of citrated blood at 1,500 rpm for 5 min and 4,000 rpm for 10 min, respectively. Plasma containing 3-4 × 10^7^ cells/cm^2^ was prepared by mixing PRP with PPP. Then, 200 *μ*L of the platelet suspension was placed on each polymer surface and incubated for 1 h at 37°C. After the films were washed three times with PBS, they were immersed in 1% glutaraldehyde in PBS for 120 min at 4°C to fix the adhered platelets. The samples were dried and sputter-coated in platinum-palladium using an ion sputter coater prior to SEM (JSM-7600FA, JEOL Ltd., Tokyo, Japan). The number of adherent platelets on the polymer films was counted in five randomly selected SEM images (*n* = 6).

### 2.7. Data Analyses

The results were analyzed using Student's *t*-test. *P* < 0.05 was used as the threshold for statistical significance between groups.

## 3. Results and Discussion

### 3.1. Characterization of Polymer Films

The surface roughness of each polymer film was analyzed by AFM (Table 1). The AFM topographical values (root mean squared; RMS) for PET, PMEA, PHEMA, and PMPC were 10, 6.7, 5.8, and 6.5 nm, respectively. The polymer-coated films were smoother than the PET film.

**Figure 1:**
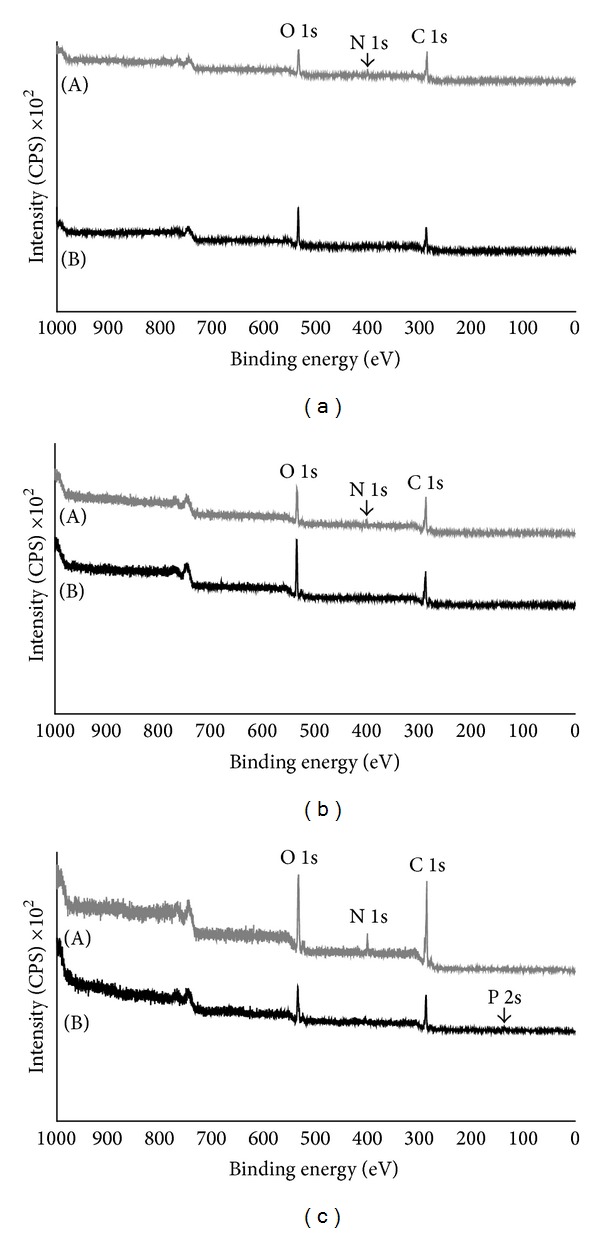
XPS spectrum of the PET film surface coated with PMEA (a), PHEMA (b), and PMPC (c). (A) indicates the XPS spectrum of the PET film surface. (B) indicates the XPS spectrum of the coated film. The atomic compositions determined from the XPS spectra match the expected composition based on the structure of each polymer.

Figure 1(a) shows the XPS spectrum of the PET film coated nitrogen-modified PET film (Figures 1(a)–1(c), line A). C 1s and O 1s peaks derived from PMEA were observed, whereas an N 1s peak for the nitrogen-modified PET film was not observed (Figure 1(a), line B). The XPS spectrum of the coated PHEMA also showed the same result (Figure 1(b)). The XPS spectra of coated PMPC also did not show an N 1s peak but showed a P 2s peak (Figure 1(c), line B). These results indicate that the PET film surface was completely covered with each polymer.

The static contact angle (*θ*) on each polymer surface was measured by the sessile drop method as shown in Table 2. The *θ* values determined by the sessile drop method for deionized water were 69° ± 2.6°, 45° ± 2.2°, 36° ± 2.7°, and 105° ± 3.3° on PET, PMEA, PHEMA, and PMPC, respectively. These data indicate that the hydrophilicity of PMEA is between that of PET and PHEMA. The static contact angle of each polymer was consistent with values in the literature [31, 32].

### 3.2. Morphology of PDL Cells on Polymer Surfaces

**Figure 2:**
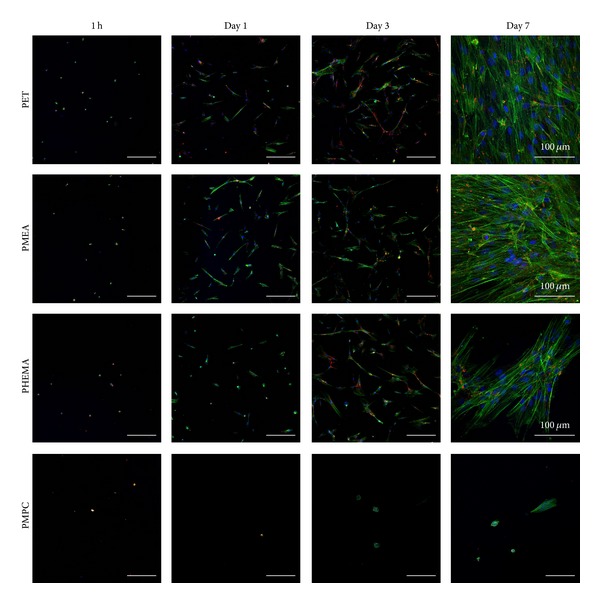
CLSM images of PDL cells cultured on polymer surfaces. Scale bars: 300 *μ*m. Blue: nucleus, green: actin, and red: vinculin. Time points are 1 h, 1 day, 3 days, and 7 days. Polymers: PET, PMEA, PHEMA, and PMPC.

Cell morphology and proliferation behavior observed by CLSM demonstrated that PDL cells adhered to PMEA and other polymer surfaces, except PMPC, with a round shape within 1 h (Figure 2). After 1 day, PDL cells had spread across the polymers. After 3 days, PDL cells had spread further and showed proliferation behavior on all polymers. After 7 days, PDL cells on PET and PMEA were confluent, and developed actin fibers were observed. PDL cells on PHEMA were not confluent but had aggregated. PDL cells did not adhere and proliferate on PMPC throughout the experiment.

### 3.3. Initial Adhesion of PDL Cells

**Figure 3:**
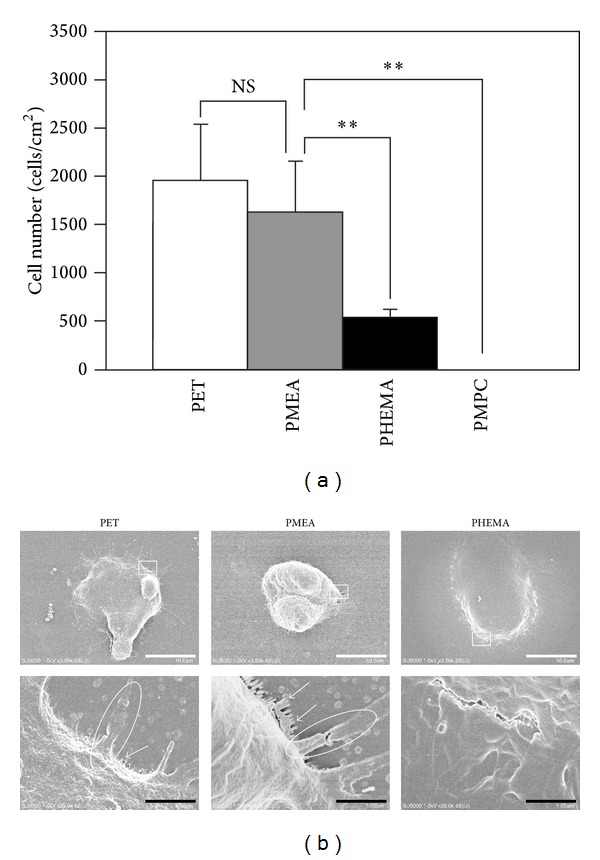
Initial adhesion of PDL cells on polymer surfaces after 1 h. (a) The number of adherent PDL cells on polymer surfaces. Polymers: PET, PMEA, PHEMA, and PMPC. **P < 0.01 versus PMEA, mean ± standard deviation, *n* = 3. (b) SEM images of PDL cells on polymer surfaces. Top scale bars: 10 *μ*m, bottom scale bars: 1.0 *μ*m. Polymers: PET, PMEA, and PHEMA. White circles indicate pseudopodia formation. White arrows indicate spike formation.

The nuclei of adherent PDL cells on each polymer surface were counted under CLSM. PDL cells adhered to PMEA and the other polymer surfaces, except for PMPC, upon incubation for 1 h (Figure 3(a)). The number of adherent PDL cells on PMEA was almost identical to that on PET and was 5 times higher than that on PHEMA.

The cell morphology observed by SEM showed differences for each polymer after 1 h (Figure 3(b)). PDL cells on PET contained some pseudopodia (white circle) and spikes (white arrow). PDL cells on PMEA contained some pseudopodia (white circle) and many spikes (white arrows). PDL cells on PHEMA contained lamellipodia. The surfaces of adherent PDL cells adherent to PET and PMEA were rougher than those on PHEMA.

### 3.4. Quantification of Projected Area, Perimeter, and Long Axis of PDL Cells

**Figure 4:**
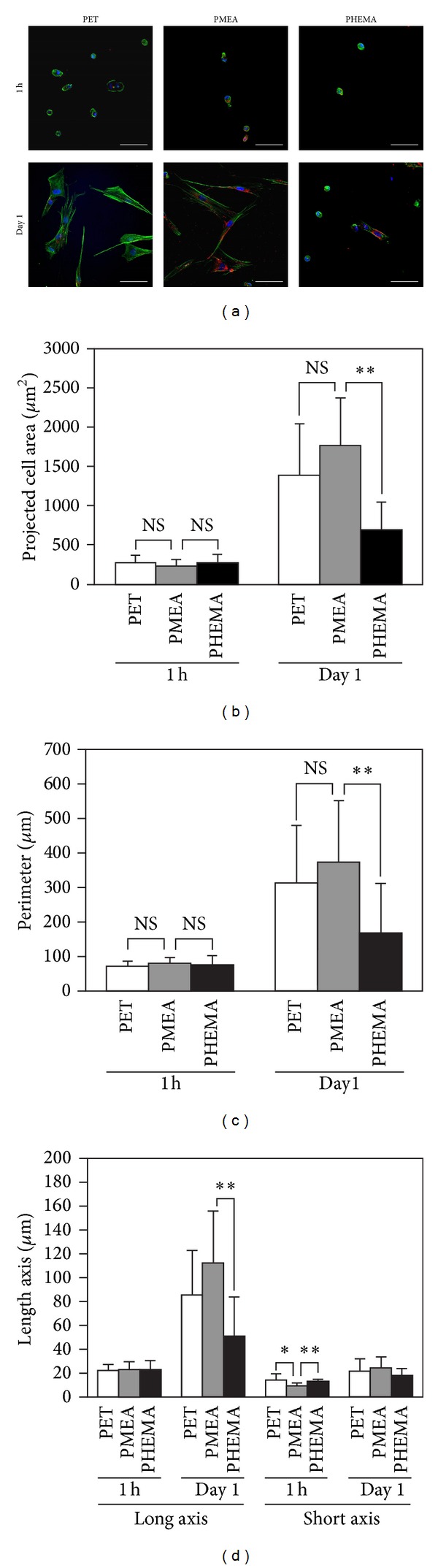
Quantification of adherent PDL cell morphologies for 1 h and 1 day. (a) CLSM images for the quantification of adherent PDL cell morphology on polymer surfaces. Scale bars: 100 *μ*m. Blue: nucleus, green: actin, and red: vinculin. Polymers: PET, PMEA, and PHEMA. (b) Projected cell area. (c) Perimeter of adherent PDL cells. (d) Long and short axes of adherent PDL cells. Polymers: PET, PMEA, and PHEMA. **P < 0.01 and *P < 0.05 versus PMEA, mean ± standard deviation, *n* = 10.

The morphology of the adherent PDL cells was quantified using CLSM images (Figure 4(a)).  Figure 4(b) shows the projected cell area on each polymer. After 1 h, the projected cell areas were 270 ± 100 *μ*m^2^, 230 ± 90 *μ*m^2^, and 270 ± 110 *μ*m^2^ on PET, PMEA, and PHEMA, respectively. After 1 day, the projected cell areas were 1390 ± 660 *μ*m^2^, 1770 ± 610 *μ*m^2^, and 690 ± 360 *μ*m^2^ on PET, PMEA, and PHEMA, respectively. The projected cell areas increased by 5.1-, 7.7-, and 2.6-fold on PET, PMEA, and PHEMA, respectively, from 1 h up to 1 day.

Figure 4(c) shows the perimeter of adherent PDL cells on each polymer. After 1 h, the perimeters were 75 ± 15 *μ*m, 80 ± 17 *μ*m, and 82 ± 27 *μ*m on PET, PMEA, and PHEMA, respectively. After 1 day, the perimeters were 300 ± 85 *μ*m, 380 ± 130 *μ*m, and 180 ± 80 *μ*m on PET, PMEA, and PHEMA, respectively. The perimeter of the adherent cells increased by 4.0-, 4.8-, and 2.2-fold on PET, PMEA, and PHEMA, respectively, from 1 h up to 1 day.

Figure 4(d) shows each axis of the adherent PDL cells on each polymer. After 1 h, the long axes (i.e., length) were 22 ± 5 *μ*m, 22 ± 7 *μ*m, and 22 ± 8 *μ*m on PET, PMEA, and PHEMA, respectively. After 1 day, the lengths were 85 ± 37 *μ*m, 110 ± 44 *μ*m, and 50 ± 33 *μ*m on PET, PMEA, and PHEMA, respectively. The length of adherent cells increased by 3.9-, 5.0-, and 2.3-fold on PET, PMEA, and PHEMA, respectively, from 1 h up to 1 day. After 1 h, the short axes (i.e., width) were 14 ± 5 *μ*m, 9 ± 2 *μ*m, and 13 ± 2 *μ*m on PET, PMEA, and PHEMA, respectively. After 1 day, the widths were 21 ± 10 *μ*m, 24 ± 9 *μ*m, and 18 ± 6 *μ*m on PET, PMEA, and PHEMA, respectively. The width of adherent cells increased by 1.5-, 2.7-, and 1.4-fold on PET, PMEA, and PHEMA, respectively, from 1 h up to 1 day. Figures 4(a)–4(d) show that PDL cells on PMEA were more spread than cells on any other polymer surface.

### 3.5. Proliferation of PDL Cells

**Figure 5:**
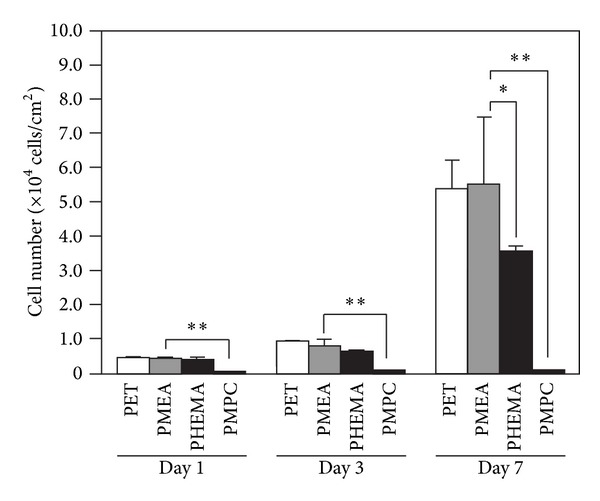
The number of PDL cells on polymer surfaces. Time points are 1 day, 3 days, and 7 days. Polymers: PET, PMEA, PHEMA, and PMPC. **P < 0.01 and *P < 0.05 versus PMEA, mean ± standard deviation, *n* = 3.

PDL cells adhered and proliferated on all polymer surfaces, except for PMPC, during the culture period (Figure 5). After 1 day and 3 days, the number of adherent PDL cells was almost identical on all of the polymer surfaces. After 7 days, the number of PDL cells on PMEA was almost the same as that on PET. The number of PDL cells on PHEMA was lower than that on PET and PMEA.

**Figure 6:**
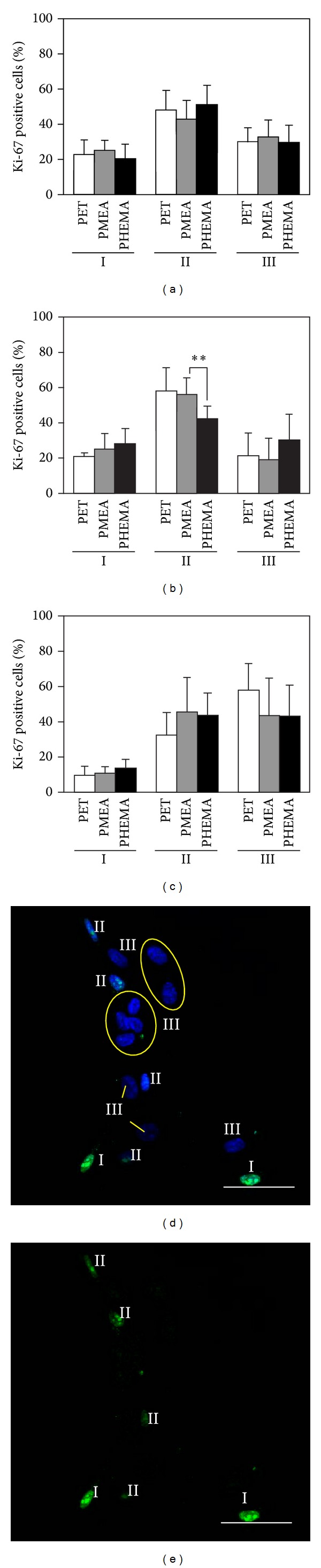
Percentage of Ki-67-positive cells on polymer surfaces: (a) 1 day, (b) 3 days, and (c) 7 days. Polymers: PET, PMEA, and PHEMA. **P < 0.01 and *P < 0.05 versus PMEA, mean ± standard deviation, *n* = 9. (d)-(e) Classification of Ki-67 staining. The images show Ki-67 staining of PDL cells on PHEMA after 3 days. Scale bars: 100 *μ*m. Blue: nucleus, green: Ki-67-positive cells. (d) CLSM images of PDL cells showing the nucleus and Ki-67 staining. (e) CLSM images of PDL cells showing Ki-67 staining. Types I and II: the Ki-67 antigen is present in the nucleus during the G1, S, and G2 phases of cell division and during mitosis. Type III: quiescent or resting cells in the G0 phase do not express the Ki-67 antigen.

Figures 6(a)–6(c) show the percentage of Ki-67-positive cells during the culture period. Ki-67-positive cells (proliferating cells; Figures 6(d)-6(e)) were categorized into types I and II. Type I showed stronger staining, while type II showed weaker staining. Ki-67-negative cells (quiescent or resting cells) were categorized as type III (Figures 6(d)-6(e)). PDL cells did not show a statistically significant difference between 1 day (Figure 6(a)) and 7 days (Figure 6(c)). After 3 days, only type II adherent PDL cells on PMEA showed a statistically significant difference relative to cells on PHEMA (Figure 6(b)), which indicates that the number of proliferating PDL cells on PMEA was higher than that on PHEMA.

### 3.6. Localization of Vinculin in Two- and Three-Dimensional Observation

**Figure 7:**
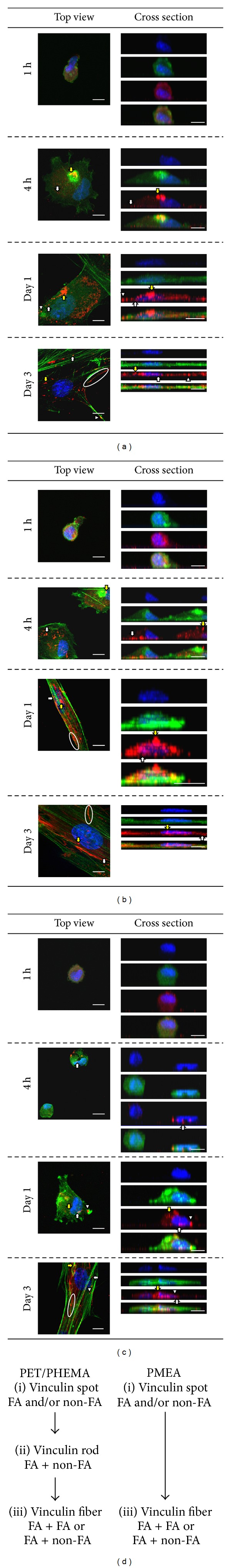
Localization of nucleus, actin, and vinculin in adherent PDL cells on polymer surfaces. Scale bars: 10 *μ*m. In the cross-section images, the top panel shows the nucleus (blue), the second panel shows the nucleus and actin (green), the third panel shows the nucleus and vinculin (red), and the bottom panel shows a merged image. The time points are 1 h, 4 h, 1 day, and 3 days. (a) PDL cells on PET. (b) PDL cells on PMEA. (c) PDL cells on PHEMA. White arrows indicate focal adhesions that were localized at the basal cell surface. Yellow arrows indicate nonfocal adhesions (non-FA) localized at the apical cell surface. White arrowheads indicate vinculin rods that were connected vertically and penetrated the adherent cell. White circles indicate vinculin fibers that were mainly oriented along the long axis of the adherent cells. (d) Schematic representation of vinculin fiber formation.

Figures 7(a)–7(c) show a top view and cross sections of adherent cells. We classified the localization of vinculin into 4 types: (i) focal adhesions (FAs) and (ii) nonfocal adhesions (non-FAs), where FAs were localized at basal cell surfaces, and non-FAs were localized at apical cell surfaces; (iii) vinculin rods that were composed of a complex of FAs and non-FAs and that were connected vertically and penetrated the cells; and (iv) vinculin fibers that were mainly oriented along the long axis of the cells.

Figure 7(a) shows PDL cells cultured on PET. After 1 h, adherent PDL cells were shaped similarly to gourds (Figure 7(a)). The cells were spherical with a thickness of approximately 15 *μ*m, and their actin and vinculin were undeveloped. After 4 h, the cells had spread to form disc-like shapes and FAs. Large spots of actin and vinculin were localized at the apical cell surfaces. The thickness of the adherent PDL cells was approximately 10 *μ*m. Vinculin was mainly localized at basal cell surfaces in FAs and apical cell surfaces in non-FAs. After 1 day, the cells were spread out thinly. Large vinculin spots were localized at apical cell surfaces in non-FAs, and FAs were also observed in addition to vinculin rods. After 3 days, the cells were spread out more thinly. Many vinculin rods were observed. FAs, non-FAs, and vinculin fibers were also observed.

Figure 7(b) shows PDL cells cultured on PMEA. After 1 h, the adherent PDL cells were shaped similarly to gourds. The cells were spherical with a thickness of approximately 11 *μ*m. Their actin and vinculin were undeveloped. After 4 h, PDL cells had spread to form disc-like shapes and FAs. Large actin spots and small vinculin spots were localized at the apical cell surfaces in non-FA. The thickness of the cells was approximately 10 *μ*m. Vinculin was mainly localized at basal cell surfaces in FAs and apical cell surfaces in non-FAs. After 1 day, the cells were spread out and spindle shaped. Large vinculin spots were localized at apical cell surfaces in non-FAs, and FAs were also observed in addition to vinculin fibers. After 3 days, the cells were spread out more thinly. FAs and non-FAs were observed, and vinculin fibers were also observed.

Figure 7(c) shows PDL cells cultured on PHEMA. After 1 h, the adherent cells were spherical with a thickness of approximately 15 *μ*m. Their actin and vinculin were undeveloped. After 4 h, the cells were slightly spread out, and they possessed FAs. After 1 day, the cells had a round shape relative to the shape of cells on the other polymers (Figures 7(a) and 7(b)). Large vinculin spots were localized at apical cell surfaces in non-FAs. FAs and vinculin rods were also observed. After 3 days, the PDL cells had spread out. Vinculin rods were also observed. FAs and non-FAs were observed in addition to vinculin fibers. The vinculin fiber-formation process is summarized in Figure 7(d). PDL cells on PMEA contained few vinculin rods, whereas PDL cells on PET and PHEMA contained many vinculin rods.

#### 3.6.1. Relationship between Cell Proliferation and Ki-67 Protein Production

As shown in Figures 2, 3(a)-3(b), 4(a)–4(d), and 5, PDL cells on PMEA showed similar adhesion and proliferation behavior to cells on PET at all-time points. The low proliferation on PHEMA was consistent with the results of Peluso et al., who found that human embryonic lung fibroblasts did not proliferate on PHEMA [33]. Based on this finding, we focused on differences in the cell cycle and quantified the percentage of Ki-67-positive cells on the polymers to identify differences in cell proliferation. As shown in Figure 6(b), the percentage of Ki-67-positive cells on PMEA at 3 days was higher than that on PHEMA. These data suggest that the higher proliferation of PDL cells on PMEA was related to the higher percentage of Ki-67-positive cells at 3 days (Figures 5 and 6(b)).

#### 3.6.2. Relationship between Vinculin Localization and Cell Proliferation

Next, we analyzed the localization of vinculin in 2 and 3 dimensions to investigate the cause of difference in the proportion of Ki-67-positive cells and to analyze cell-material interactions. Our results suggest that PDL cells on PMEA have stronger PDL cell-material interactions than cells on PHEMA because cells on PMEA exhibited high vinculin localization and Ki-67 protein production. In our next study, we will attempt to elucidate the influence of the chemical structure of the synthetic polymer on cell behavior by altering the composition of the main chain and/or the terminal functional group of the side chain.

As shown in Figures 7(a)–7(d), we observed some unique vinculin localization in non-FAs, vinculin rods, and vinculin fibers. Kanchanawong et al. reported that focal adhesions link the extracellular matrix to the actin cytoskeleton, and vinculin localized in focal adhesions probably links integrin to actin directly, as the distribution of vinculin is consistent with its binding to sites along the talin rod domain and actin, which may serve to buttress the integrin-talin-actin linkage [29]. Therefore, vinculin localization was expected to occur at basal cell surfaces contacting the materials; however, we observed that vinculin localized in non-FAs at apical cell surfaces and in vinculin rods that were composed of a complex of FAs and non-FAs that were connected vertically and penetrated the cells (Figures 7(a)–7(d)). In addition, we observed that vinculin fibers appeared to be similar to the supermaturation of focal adhesions reported by Dugina et al., who showed that increased extradomain A fibronectin expression induced by transforming growth factor *β* (TGF*β*) was accompanied by *α*-smooth muscle actin expression and focal adhesion supermaturation in fibroblasts [34]. The non-FAs probably link extracellular matrix proteins such as fibronectin with the apical cell surface. In future studies, we will evaluate the role of the unique vinculin localization in non-FAs, vinculin rods, and vinculin fibers with regard to cell behavior.

### 3.7. Comparison of Platelet and PDL Cell Adhesion on PMEA and PMPC

**Figure 8:**
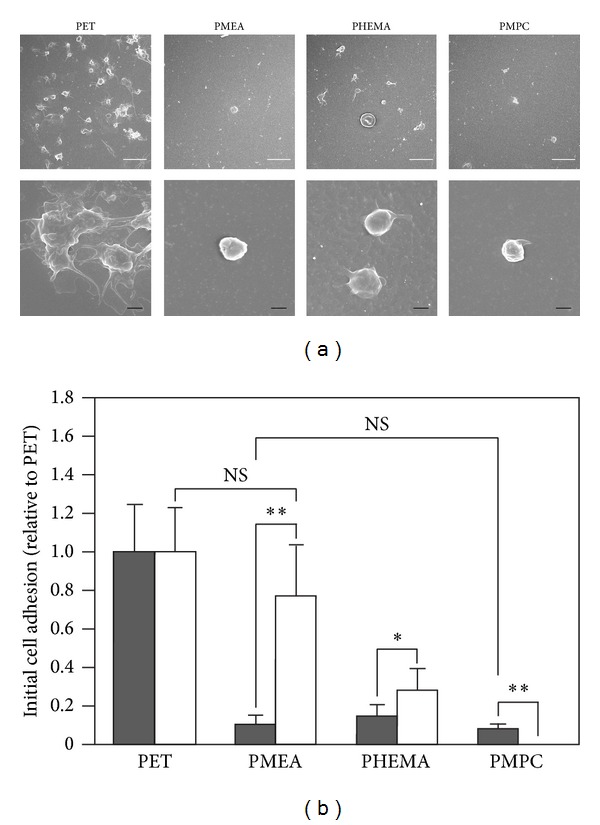
Comparison of platelet and PDL cell adhesion after 1 h. (a) SEM images of adherent platelets on polymer surfaces. Top scale bars: 10 *μ*m, bottom scale bars: 1.0 *μ*m. Polymers: PET, PMEA, PHEMA, and PMPC. (b) Comparison of platelet and PDL cell adhesion after 1 h. Gray bar indicates platelet adhesion and white bar indicates PDL cell adhesion, respectively, relative to PET. Polymers: PET, PMEA, PHEMA, and PMPC. **P < 0.01 and *P < 0.05 versus PET, mean ± standard deviation, platelets: *n* = 6, PDL cells: *n* = 9.

Figure 8(a) shows the adherent platelets on each polymer as determined by SEM.  Figure 8(b) shows the number of adherent platelets and PDL cells on each polymer relative to that on PET. Platelet adhesion on PMEA and PMPC was low, and the number of platelets on PMEA was not significantly different from that on PMPC. Many adherent PDL cells were observed on PMEA and PET, whereas relatively few adherent PDL cells were observed on PHEMA and PMPC. PDL cells adhered to PMEA, whereas platelets hardly adhered to PMEA. In contrast, both platelets and PDL cells did not adhere to PMPC.

#### 3.7.1. Relationship between Biocompatibility and Cell Adhesions

Conventional synthetic biocompatible polymers such as PMPC, poly(sulfobetaine methacrylate), poly(carboxybetaine methacrylate), and poly(ethylene glycol) (PEG) are known to demonstrate low protein adsorption and/or no platelet adhesion [35–39]. PDL cells hardly adhered and proliferated on PMPC, which was consistent with the findings of a previous study by Iwasaki et al., who reported that adhesion of human promyelocytic leukemia cells and human uterine cervical cancer cells was completely suppressed on PMPC [36]. We previously reported that, we previously reported that PMEA has excellent biocompatibility with human blood cells [15]. As shown in Figures 8(a)-8(b), the number of platelets on PMEA was not significantly different from the number of platelets on PMPC. In contrast, PDL cells adhered to PMEA, and the number of PDL cells on PMEA was higher than that on PHEMA and PMPC (Figure 8(b)). These findings raise the question of why PDL cells adhere to biocompatible PMEA but platelets do not, especially when both platelets and PDL cells do not adhere to PMPC in a limited manner. Although we have no clear evidence to answer this question or to explain why PDL cells demonstrate higher growth rates on PMEA, we can offer the following speculations in terms of the 3 steps required for cell adhesion on polymer surfaces.

#### 3.7.2. Relationship between Adsorbed Proteins and Cell Adhesion

Initially, when a polymer surface comes in contact with cell culture medium, it absorbs water, and a specific water structure is formed on the polymer surface [21]. On PET or PHEMA, the absorbed water creates a nonfreezing water layer and a free water layer. We have also reported that PMEA and PMPC form another layer called the intermediate water layer [20, 23, 40].

Proteins in the cell culture medium then adsorb to the water layer. When proteins adsorb to the nonfreezing water layer on polymer surfaces such as PET and PHEMA, a strong conformational change of the adsorbed proteins occurs, and many cell-binding sites are exposed. Intermediate water in PMEA does not induce a conformational change in the adsorbed proteins (bovine serum albumin and fibrinogen), and, thus, potential platelet-binding sites are minimally exposed [15, 20].

Finally, platelets and PDL cells adhere to the cell-binding sites of the adsorbed proteins. We recently found that adsorption-induced deformation of fibrinogen (platelet adhesion ligand), which is required for the adhesion of platelets, does not occur on PMEA [41]. In contrast, fibronectin (PDL cell adhesion ligand) was deformed on PMEA [41]. Therefore, we concluded that PDL cells and not platelets are capable of adhering to PMEA based on this protein deformation difference between polymer films. We suppose that the existence of an intermediate water layer alters the amount of exposed cell-binding sites, which results in differing cell adhesion on each polymer.

#### 3.7.3. Relationship between Biocompatibility and Intermediate Water

In addition, we have reported that hydrated PMPC and PEG, as well as various proteins and polysaccharides that are well-known biocompatible polymers, contain intermediate water [20, 23, 42, 43]. In contrast, poorly biocompatible polymers do not contain intermediate water [31]. Free water has high mobility and is unable to shield the polymer surface or the nonfreezing water layer on the polymer surface [23]. Because intermediate water is weakly bound to the polymer molecules or to nonfreezing water, it forms a more stable structure than free water [23]. Based on these findings, we hypothesized that intermediate water, which prevents proteins and platelets from directly contacting the polymer surface or nonfreezing water on the polymer surface, plays an important role in biocompatibility and cell adhesion [23], and the amount of intermediate water affects protein adsorption and cell adhesion [20, 44, 45].

#### 3.7.4. Relationship between Intermediate Water Content and Cell Adhesion

Intermediate water content in hydrated PMEA (4.5 wt%) [31] prevented platelet adhesion but did not prevent PDL cell adhesion. In contrast, higher intermediate water content in hydrated PMPC (28.5 wt%) [40] prevented the adhesion of both platelets and PDL cells. The value of the intermediate water content in hydrated PMPC reflects the value for the MPC homopolymer because Kitano et al. confirmed that the MPC-rich domain is directed toward the surface in water [32]. We assume that the higher intermediate water content in hydrated PMPC relative to PMEA prevented cell adhesion because the thick intermediate water layer may have shielded the polymer surface or nonfreezing water layer. In contrast, the thin intermediate water layer in hydrated PMEA likely prevented platelet adhesion but did not prevent PDL cell adhesion.

#### 3.7.5. Relationship between Cell Properties and Cell Adhesion

We also consider the possibility that cell characteristics affected cell adhesion. Platelets are floating cells and mainly adhere to surfaces via glycoprotein IIb/IIIa [46]. PDL cells are anchorage-dependent and mainly adhere to surfaces via integrin *α*_5_ *β*_1_ [47]. Furthermore, differences of cell size and weight are present, as PDL cells are larger (10–15 *μ*m) and heavier than platelets (2–4 *μ*m); therefore, PDL cells may demonstrate increased adhesion simply because of their weight. In our next study, we will attempt to clarify the molecular mechanisms underlying cell adhesion on PMEA with regard to the intermediate water content and cell adhesion.

### 3.8. PMEA: Applications for Tissue Engineering Scaffolds in a Blood-Rich Environment

**Figure 9:**
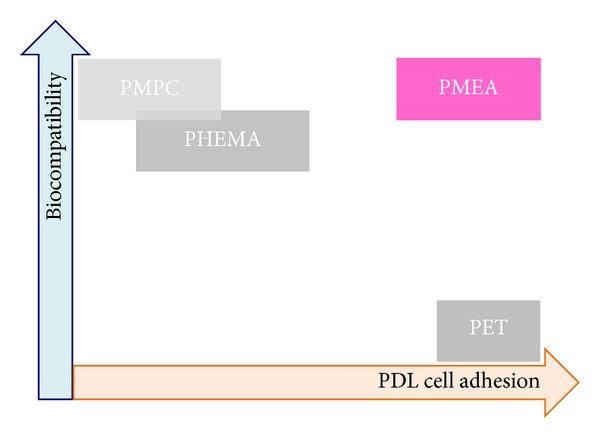
Relationship between PDL cell adhesion and biocompatibility on PET, PMEA, PHEMA, and PMPC.

As shown in Figures 2, 3(a), 5, and 8(b), PDL cells adhered to and proliferated on PET, whereas PET was not biocompatible for human platelets. PDL cells on PHEMA proliferated but had aggregated (Figures 2 and 5), and PHEMA was thus also found to be nonbiocompatible for tissue engineering using PDL cells. As shown in Figures 2, 3(a), 5, and 8(b), PDL cells adhered and proliferated on biocompatible PMEA without platelet adhesion; however, PDL cells did not adhere and proliferate on biocompatible PMPC. PMEA and PMPC have been previously identified as blood-compatible (nonplatelet-adhesive) polymers. However, recent advances in medicine require the use of blood-compatible polymers that do not exhibit blood cell attachment to isolate stem cells from blood. Our results challenge the widely accepted notion that biocompatible (blood-compatible) polymers (such as PMPC) do not permit cell adhesion as shown in Figure 9. We observed PDL cell adhesion on the biocompatible (blood-compatible) polymer PMEA in the absence of incorporated, substrate-bound, cell-adhesive ligands and antibodies. We therefore consider that PMEA could be used in smart biomaterials. Different cell types may thus be selected by PMEA based on differences in cell adhesion strength. It should be noted that PMEA has been approved by the FDA and can be used in a blood-rich environment. Therefore, biocompatible PMEA may provide an excellent scaffold for tissue engineering using PDL cells in humans as well as for culturing tissue-derived cells in a blood-rich environment.

## 4. Conclusion

We found that PDL cells, but not platelets, adhered to biocompatible PMEA. We also observed unique vinculin localization in non-FAs, vinculin rods, and vinculin fibers. In addition, PDL cells on PMEA proliferated better than those on PHEMA. Therefore, PMEA may provide an excellent scaffold material for tissue engineering using PDL cells in humans and also for culturing tissue-derived cells in a blood-rich environment.

## Supplementary Material

Supplementary Material Figure. 1. Chemical structures of poly(2-methoxyethyl acrylate) (PMEA) (a), poly(2-hydroxyethyl methacrylate) (PHEMA) (b), and poly[(2-methacryloyloxyethyl phosphorylcholine)-co-(n-butyl methacrylate)] (PMPC) (c) n:m = 30:70.

## Figures and Tables

**Table 1:** AFM topographical data. RMS: root mean squared roughness. Scan size 10 × 10 *μ*m^2^.

Polymer	RMS (nm)
PET	10
PMEA	6.7
PHEMA	5.8
PMPC	6.5

**Table 2:** Static contact angles of polymer surfaces.

Polymer	Sessile drop (degrees), (±SD)
PET	69.2 (±2.6)
PMEA	45.0 (±2.2)
PHEMA	36.0 (±2.7)
PMPC	105.2 (±3.3)
