# Supplementary material for: Adhesion and Proliferation of Human Periodontal Ligament Cells on Poly(2-methoxyethyl acrylate)
Source: Biomed Res Int. 2014 Aug 6;2014:102648. doi: 10.1155/2014/102648 (PMC4140152; doi:10.1155/2014/102648)
Supplement: Supplementary file 1 — Supplementary Material Figure. 1. Chemical structures of poly(2-methoxyethyl acrylate) (PMEA) (a), poly(2-hydroxyethyl methacrylate) (PHEMA) (b), and poly[(2-methacryloyloxyethyl phosphorylcholine)-co-(n-butyl methacrylate)] (PMPC) (c) n:m = 30:70. [file 102648.f1.pdf]

Supplementary Material

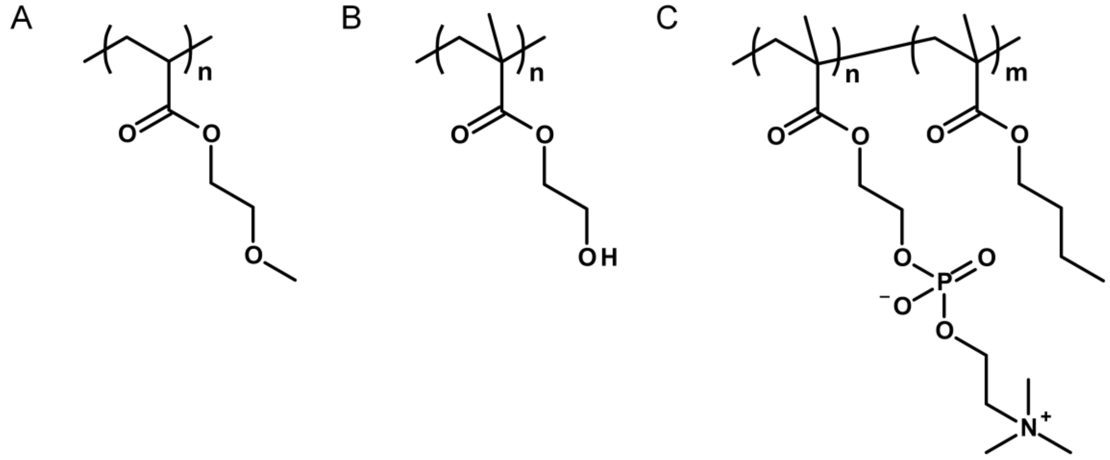

Supplementary data Fig. 1. Chemical structures of poly(2-methoxyethyl acrylate) (PMEA) (**A**), poly(2-hydroxyethyl methacrylate) (PHEMA) (**B**), and poly[(2-methacryloyloxyethyl phosphorylcholine)-*co*-(*n*-butyl methacrylate)] (PMPC) (**C**) n:m = 30:70.
